# Supplementary material for: Multiscale three-dimensional surface reconstruction and surface roughness of porcine left anterior descending coronary arteries
Source: R Soc Open Sci. 2019 Sep 11;6(9):190915. doi: 10.1098/rsos.190915 (PMC6774942; doi:10.1098/rsos.190915)
Supplement: Original Dataset [file rsos190915supp1.pdf]

Alicona

100x

RaC

284m	*285m*	*286m*	*296m*	*297m*	*298m*
3.14E-07	8.30E-07	2.43E-07	5.03E-07	1.23E-06	8.61E-07
3.26E-07	4.85E-07	4.54E-07	5.28E-07	4.20E-07	6.29E-07
3.35E-07	6.07E-07	4.02E-07	4.97E-07	1.03E-06	5.42E-07
3.72E-07	5.64E-07	1.07E-07	3.39E-07	1.25E-06	4.92E-07
2.18E-07	4.12E-07	2.94E-07	4.54E-07	1.23E-06	5.64E-07
**3.13E-07**	**5.79E-07**	**3.00E-07**	**4.64E-07**	**1.03E-06**	**6.18E-07**
**5.12E-08**	**1.42E-07**	**1.22E-07**	**6.69E-08**	**3.17E-07**	**1.30E-07**

RaL

284m	*285m*	*286m*	*296m*	*297m*	*298m*
1.66E-07	1.62E-07	1.64E-07	2.45E-07	1.96E-07	1.53E-07
7.27E-08	4.62E-07	1.31E-07	2.24E-07	6.13E-07	1.65E-07
1.09E-07	2.86E-07	1.87E-07	2.26E-07	4.75E-07	2.71E-07
9.36E-08	1.65E-07	1.19E-07	2.59E-07	7.46E-07	3.45E-07
2.46E-07	2.01E-07	1.77E-07	2.10E-07	4.31E-07	6.02E-07
**1.37E-07**	**2.55E-07**	**1.56E-07**	**2.33E-07**	**4.92E-07**	**3.07E-07**
**6.26E-08**	**1.12E-07**	**2.61E-08**	**1.72E-08**	**1.85E-07**	**1.64E-07**

50x

RaC

284m	*285m*	*286m*	*296m*	*297m*	*298m*
4.91E-07	6.03E-07	6.57E-07	3.74E-07	1.49E-06	5.93E-07
5.40E-07	6.14E-07	8.78E-07	4.56E-07	1.48E-06	7.75E-07
4.38E-07	8.18E-07	8.72E-07	3.94E-07	1.18E-06	4.99E-07
4.00E-07	6.07E-07	6.94E-07	3.52E-07	7.72E-07	5.17E-07
3.23E-07	5.46E-07	6.27E-07	3.01E-07	1.90E-06	4.15E-07
**4.38E-07**	**6.38E-07**	**7.46E-07**	**3.75E-07**	**1.37E-06**	**5.60E-07**
**7.47E-08**	**9.35E-08**	**1.08E-07**	**5.07E-08**	**3.76E-07**	**1.21E-07**

RaL

284m	*285m*	*286m*	*296m*	*297m*	*298m*
1.59E-07	2.19E-07	2.39E-07	2.59E-07	3.46E-07	1.82E-07
2.87E-07	2.25E-07	1.63E-07	2.53E-07	7.64E-07	1.74E-07
1.21E-07	3.41E-07	2.04E-07	1.92E-07	3.79E-07	1.79E-07
2.28E-07	4.13E-07	1.48E-07	3.25E-07	3.08E-07	1.97E-07
1.77E-07	2.46E-07	2.02E-07	1.54E-07	3.74E-07	3.74E-07
**1.95E-07**	**2.89E-07**	**1.91E-07**	**2.37E-07**	**4.34E-07**	**2.21E-07**
**5.75E-08**	**7.61E-08**	**3.24E-08**	**5.89E-08**	**1.67E-07**	**7.70E-08**

20x

RaC

284m	*285m*	*286m*	*296m*	*297m*	*298m*
3.93E-07	7.65E-07	8.53E-07	3.52E-07	1.23E-06	4.88E-07
3.13E-07	7.94E-07	9.76E-07	4.66E-07	1.33E-06	5.70E-07
3.26E-07	6.88E-07	1.02E-06	3.97E-07	1.18E-06	7.29E-07
4.67E-07	5.90E-07	6.99E-07	3.73E-07	9.77E-07	6.41E-07
7.42E-07	7.37E-07	5.27E-07	4.39E-07	1.74E-06	8.74E-07
**4.48E-07**	**7.15E-07**	**8.16E-07**	**4.05E-07**	**1.29E-06**	**6.60E-07**
**1.57E-07**	**7.16E-08**	**1.83E-07**	**4.19E-08**	**2.51E-07**	**1.33E-07**

RaL

284m	*285m*	*286m*	*296m*	*297m*	*298m*
1.71E-07	2.16E-07	1.69E-07	2.11E-07	3.56E-07	3.05E-07
1.70E-07	2.43E-07	3.07E-07	2.28E-07	4.67E-07	2.70E-07
1.52E-07	1.87E-07	1.88E-07	2.72E-07	3.25E-07	2.42E-07
1.74E-07	2.79E-07	1.92E-07	2.38E-07	3.52E-07	9.47E-08
2.55E-07	2.48E-07	2.93E-07	2.12E-07	4.76E-07	1.62E-07
**1.85E-07**	**2.35E-07**	**2.30E-07**	**2.32E-07**	**3.95E-07**	**2.15E-07**
**3.61E-08**	**3.12E-08**	**5.78E-08**	**2.22E-08**	**6.35E-08**	**7.64E-08**

10x

RaC

284m	*285m*	*286m*	*296m*	*297m*	*298m*
6.73E-07	6.01E-07	8.31E-07	1.20E-06	2.13E-06	5.96E-07
6.23E-07	1.21E-06	1.11E-06	1.06E-06	1.31E-06	7.23E-07
4.50E-07	1.01E-06	1.28E-06	1.06E-06	9.93E-07	8.77E-07
3.93E-07	4.62E-07	7.82E-07	4.81E-07	8.77E-07	8.78E-07
3.85E-07	9.85E-07	1.15E-06	4.09E-07	1.28E-06	1.42E-06
**5.05E-07**	**8.52E-07**	**1.03E-06**	**8.41E-07**	**1.32E-06**	**8.99E-07**
**1.20E-07**	**2.76E-07**	**1.90E-07**	**3.28E-07**	**4.38E-07**	**2.82E-07**

RaL

284m	*285m*	*286m*	*296m*	*297m*	*298m*
2.29E-07	2.43E-07	2.93E-07	5.03E-07	4.65E-07	2.49E-07
2.41E-07	3.82E-07	3.11E-07	2.63E-07	3.40E-07	2.65E-07
2.50E-07	3.47E-07	3.09E-07	3.44E-07	4.01E-07	4.59E-07
2.91E-07	2.80E-07	3.48E-07	2.40E-07	5.26E-07	5.83E-07
2.41E-07	3.19E-07	3.87E-07	4.34E-07	6.62E-07	2.29E-07
**2.50E-07**	**3.14E-07**	**3.30E-07**	**3.57E-07**	**4.79E-07**	**3.57E-07**
**2.15E-08**	**4.90E-08**	**3.37E-08**	**9.99E-08**	**1.11E-07**	**1.40E-07**

SEM

100x

RaC

296m	*297m*	*298m*	*287m*	*289m*
2.15E-07	2.08E-07	1.24E-07	2.27E-07	4.40E-07
2.45E-07	1.16E-07	1.43E-07	3.54E-07	3.41E-07
2.23E-07	1.21E-07	8.50E-08	2.04E-07	2.89E-07
1.07E-07	1.79E-07	5.90E-08	3.60E-07	9.70E-08
1.34E-07	1.31E-07	8.90E-08	2.11E-07	1.81E-07
**1.85E-07**	**1.51E-07**	**1.00E-07**	**2.71E-07**	**2.70E-07**
**5.41E-08**	**3.62E-08**	**2.98E-08**	**7.05E-08**	**1.20E-07**

RaL

296m	*297m*	*298m*	*287m*	*289m*
2.98E-07	2.22E-07	7.20E-08	1.88E-07	1.70E-07
3.14E-07	3.01E-07	1.31E-07	1.64E-07	2.06E-07
1.40E-07	9.90E-08	2.47E-07	1.14E-07	6.00E-08
1.34E-07	2.89E-07	1.44E-07	1.23E-07	3.57E-07
1.47E-07	3.58E-07	8.80E-08	8.00E-08	1.81E-07
**2.07E-07**	**2.54E-07**	**1.36E-07**	**1.34E-07**	**1.95E-07**
**8.14E-08**	**8.87E-08**	**6.13E-08**	**3.81E-08**	**9.53E-08**

1000x

RaC

296m	*297m*	*298m*	*287m*	*289m*	*284p*	*285p*	*286p*	*296p*	*297p*	*298p*	*284d*	*285d*	*286d*	*296d*	*297d*	*298d*
1.60E-07	3.50E-07	2.80E-07	3.10E-07	1.00E-07	4.00E-08	1.90E-07	2.70E-07	2.80E-07	2.80E-07	1.50E-07	4.60E-07	1.20E-07	3.50E-07	2.30E-07	2.10E-07	1.70E-07
2.90E-07	4.00E-07	5.40E-07	3.40E-07	9.00E-08	3.00E-08	2.20E-07	4.50E-07	1.80E-07	3.00E-07	4.00E-07	3.20E-07	1.80E-07	8.10E-07	1.80E-07	1.90E-07	1.30E-07
1.30E-07	5.30E-07	2.40E-07	4.50E-07	1.10E-07	3.00E-08	2.00E-07	3.80E-07	1.60E-07	2.00E-07	1.80E-07	4.10E-07	1.40E-07	4.60E-07	3.00E-07	2.60E-07	1.50E-07
1.80E-07	3.30E-07	3.80E-07	2.30E-07	1.80E-07	5.00E-08	2.30E-07	2.70E-07	2.90E-07	3.20E-07	2.70E-07	6.30E-07	1.40E-07	3.80E-07	2.40E-07	2.00E-07	2.10E-07
1.70E-07	3.00E-07	1.50E-07	2.20E-07	2.20E-07	5.00E-08	1.50E-07	2.80E-07	2.00E-07	2.50E-07	2.10E-07	3.70E-07	1.30E-07	3.50E-07	1.70E-07	1.40E-07	7.00E-08
**1.86E-07**	**3.82E-07**	**3.18E-07**	**3.10E-07**	**1.40E-07**	**4.00E-08**	**1.98E-07**	**3.30E-07**	**2.22E-07**	**2.70E-07**	**2.42E-07**	**4.38E-07**	**1.42E-07**	**4.70E-07**	**2.24E-07**	**2.00E-07**	**1.46E-07**
**5.46E-08**	**8.08E-08**	**1.33E-07**	**8.37E-08**	**5.10E-08**	**8.94E-09**	**2.79E-08**	**7.29E-08**	**5.31E-08**	**4.20E-08**	**8.84E-08**	**1.06E-07**	**2.04E-08**	**1.75E-07**	**4.67E-08**	**3.85E-08**	**4.63E-08**

RaL

296m	*297m*	*298m*	*287m*	*289m*	*284p*	*285p*	*286p*	*296p*	*297p*	*298p*	*284d*	*285d*	*286d*	*296d*	*297d*	*298d*
1.90E-07	3.50E-07	1.70E-07	5.10E-07	2.30E-07	2.00E-08	2.80E-07	3.70E-07	3.60E-07	3.10E-07	2.30E-07	2.50E-07	1.60E-07	5.20E-07	2.50E-07	4.00E-07	1.40E-07
8.00E-08	1.50E-07	4.00E-07	5.70E-07	2.00E-07	4.00E-08	1.60E-07	5.40E-07	2.90E-07	2.80E-07	2.70E-07	5.60E-07	1.70E-07	7.00E-07	1.40E-07	2.80E-07	1.20E-07
1.10E-07	2.60E-07	2.60E-07	2.90E-07	1.20E-07	4.00E-08	3.00E-07	4.30E-07	2.10E-07	2.40E-07	3.80E-07	6.90E-07	1.20E-07	4.40E-07	2.00E-07	1.80E-07	1.20E-07
1.50E-07	3.40E-07	4.10E-07	2.30E-07	1.90E-07	4.00E-08	2.20E-07	2.60E-07	3.80E-07	2.20E-07	2.10E-07	2.70E-07	1.90E-07	8.10E-07	1.40E-07	2.10E-07	1.90E-07
8.00E-08	2.10E-07	2.60E-07	3.30E-07	5.00E-08	7.00E-08	2.30E-07	2.20E-07	2.20E-07	2.90E-07	1.90E-07	4.50E-07	1.00E-07	8.50E-07	1.40E-07	3.60E-07	1.10E-07
**1.22E-07**	**2.62E-07**	**3.00E-07**	**3.86E-07**	**1.58E-07**	**4.20E-08**	**2.38E-07**	**3.64E-07**	**2.92E-07**	**2.68E-07**	**2.56E-07**	**4.44E-07**	**1.48E-07**	**6.64E-07**	**1.74E-07**	**2.86E-07**	**1.36E-07**
**4.26E-08**	**7.63E-08**	**9.19E-08**	**1.31E-07**	**6.49E-08**	**1.60E-08**	**4.92E-08**	**1.16E-07**	**6.97E-08**	**3.31E-08**	**6.74E-08**	**1.68E-07**	**3.31E-08**	**1.60E-07**	**4.45E-08**	**8.43E-08**	**2.87E-08**

2000x

RaC

296m	*297m*	*298m*	*287m*	*289m*
1.90E-07	1.50E-07	2.10E-07	1.50E-07	6.00E-08
1.80E-07	6.00E-08	3.90E-07	1.20E-07	7.00E-08
1.30E-07	1.30E-07	4.00E-07	1.50E-07	1.00E-07
8.00E-08	1.20E-07	3.50E-07	2.60E-07	1.70E-07
1.10E-07	6.00E-08	2.60E-07	3.80E-07	1.00E-07
**1.38E-07**	**1.04E-07**	**3.22E-07**	**2.12E-07**	**1.00E-07**
**4.17E-08**	**3.72E-08**	**7.47E-08**	**9.66E-08**	**3.85E-08**

RaL

296m	*297m*	*298m*	*287m*	*289m*
1.00E-07	5.00E-08	2.30E-07	1.90E-07	8.00E-08
1.10E-07	1.00E-07	1.30E-07	2.00E-07	8.00E-08
2.70E-07	7.00E-08	2.70E-07	3.10E-07	9.00E-08
9.00E-08	2.10E-07	1.50E-07	8.00E-08	1.40E-07
2.50E-07	1.20E-07	2.50E-07	1.30E-07	1.00E-07
**1.64E-07**	**1.10E-07**	**2.06E-07**	**1.82E-07**	**9.80E-08**
**7.89E-08**	**5.55E-08**	**5.57E-08**	**7.73E-08**	**2.23E-08**

AFM

		Sample														
		1					2					3				
		1 (y=244),(x=86)	2 (y=384),(x=414)	3 (y=97), (x=681)	Avg	SD	1 (y=109), (x=80)	2 (y=342), (x=426)	3 (y=557), (x=717)	Avg	SD	1 (y=142), (x=46)	2 (y=361), (x=339)	3 (y=593), (x=806)	Avg	SD
Horzizontal	Avg Height (m)	3.19E-16	9.11E-18	5.92E-16	3.07E-16	2.38E-16	2.09E-16	2.04E-16	3.08E-16	2.40E-16	4.81E-17	4.76E-16	3.00E-16	2.76E-16	3.50E-16	8.92E-17
	Ra (nm)	592.50	555.60	592.40	580.17	17.37	366.40	366.40	599.50	444.10	109.88	420.4	441.6	572	478.00	67.03
	Rq (nm)	671.30	690.60	735.90	699.27	27.08	460.60	460.60	702.20	541.13	113.89	521.2	563.7	653.2	579.37	55.02
	Rt (um)	2.610	3.921	3.378	3.303	0.538	3.259	3.259	4.042	3.520	0.369	2.533	3.313	2.580	2.809	0.357
Vertical	Avg Height (nm)	201.70	573.00	270.50	348.40	161.28	499.20	481.70	276.70	419.20	101.02	169.80	31.45	93.72	98.32	56.57
	Ra (nm)	308.90	319.70	304.50	311.03	6.39	279.20	266.10	453.60	332.97	85.47	390.70	491.90	292.40	391.67	81.45
	Rq (nm)	387.70	434.70	428.70	417.03	20.89	381.80	348.70	601.70	444.07	112.28	568.10	641.00	394.80	534.63	103.26
	Rt (um)	1.905	2.472	1.913	2.097	0.265	2.868	1.818	3.288	2.658	0.618	4.299	2.628	1.846	2.924	1.023
Whole Image	Avg Height (m)	2.43E-16					2.61E-16					8.57E-17				
	Ra (nm)	630.50					512.50					436.7				
	Rq (nm)	766.20					630.50					556.1				
	Rt (um)	6.640					5.049					5.623				

	Dimensions	Area for roughness	
		H	V
Sample 1	49.76*24.76	49.76*2.39	2.39*24.76
Sample 2	50*33.74	50*2.39	2.39*33.74
Sample 3	51.76*37.66	51.76*2.39	2.39*37.66
